# Is there a "weekend effect" in kidney transplantation?

**DOI:** 10.1371/journal.pone.0190227

**Published:** 2017-12-28

**Authors:** Katharina Schütte-Nütgen, Gerold Thölking, Maximilian Dahmen, Felix Becker, Linus Kebschull, René Schmidt, Hermann Pavenstädt, Barbara Suwelack, Stefan Reuter

**Affiliations:** 1 Department of Internal Medicine D, Division of General Internal Medicine, Nephrology and Rheumatology, University Hospital Münster, Münster, Germany; 2 Department of General and Visceral Surgery, University Hospital Münster, Münster, Germany; 3 Institute of Biostatistics and Clinical Research, University Hospital Münster, Münster, Germany; University of Toledo, UNITED STATES

## Abstract

The ‘weekend effect’ describes increased adverse outcomes after weekend hospitalization.

We examined weekend-weekday differences in the outcome of 580 patients following renal transplantation (RTx, brain dead donors) between January 2007 and December 2014 at our center. 3-year patient and graft survival, incidence of delayed graft function (DGF), acute rejections and estimated glomerular filtration rate (eGFR, CKD-EPI) at 1 year as well as surgical complications were assessed. Of all 580 transplants, 416 (71.7%) were performed on weekdays (Monday-Friday) and 164 (28.3%) on weekends (Saturday-Sunday). 3-year patient and graft survival, frequencies of DGF, acute rejections and 1-year eGFR as well as length of hospital stay were similar between RTx patients transplanted on weekdays or weekends, respectively. However, a noticeable difference was detected with regard to surgical complications which were more frequent in RTx patients transplanted on weekends. All results remained consistent across all definitions of weekend status. Our results suggest that weekend transplant status does not affect functional short-term and long-term outcomes after RTx. The standardized protocols and operationalized processes applied in RTx might contribute to this finding and may provide a model for other medical procedures that are performed on weekends to improve efficiency and outcomes. The higher rate of surgical complications after weekend RTx needs further elaboration to fully assess the presence of a weekend effect in RTx.

## Introduction

The time point of renal transplantation (RTx) with kidney grafts from brain death donors is unpredictable and determined by diagnosis of brain death, decision about multiorgan donors, allocation, organ transport and recipient preparation [1,2]. Cold ischaemia time is associated with an increased risk of delayed graft function (DGF), acute rejection and even increased mortality [3–5]. Therefore, RTx is frequently performed as an emergency surgery on weekends to avoid unnecessary prolongation of cold ischaemia time. However, numerous studies have shown that hospital admission on weekends is associated with increased mortality and adverse events compared to weekday admission. This so-called ‘weekend effect’ has been described for various medical and surgical conditions such as myocardial infarction, ruptured aortic aneurysm, pulmonary embolism or stroke [6–8]. Different reasons to explain the weekend effect have been postulated like reduced or less-experienced medical staff, differences in the delivery or quality of care and severity of illness of patients admitted to hospital [9,10].

Recent data from the US and UK did not show a weekend effect for kidney transplant patients [11,12]. However, though definite outcome parameters such as patient and allograft survival were analyzed in these studies, other graft specific outcomes such as allograft function determined by estimated glomerular filtration rate (eGFR), episodes of acute rejections or postoperative surgery complications which can significantly influence the outcome after RTx and also constitute a relevant health care-associated cost factor were not investigated. As the incidence of nonsurgery-related graft losses is decreased by advanced immunosuppressive regimens, the shortage of organ donations is ongoing and offered kidneys often hold severe arteriosclerosis, especially surgical performance can significantly contribute to allograft survival [13].

Considering published media reports of UK patients refusing transplant operations on Saturday or Sundays to avoid the weekend effect and the recent alerting finding by Mohan et al. who reported that a weekend effect does exist regarding the discard rates of deceased donor kidneys in the US [11,14], further examination to alleviate patient concern and to guide strategic planning of kidney transplant services is needed.

To date, no study exists that has investigated a potential weekend effect in German transplant centers or any other Eurotransplant center. As transplant policies and health care systems in the US and UK are different from Germany we aimed to analyze whether transplant outcomes were similar for weekday versus weekend transplantations in a single center study at the University Hospital Muenster. In addition to patient and graft survival, secondary outcome parameters such as kidney function, acute rejections and surgical complications as well as length of hospital stay were investigated.

## Patients and methods

### Patients

Prior to analysis, data of all patients were anonymized and de-identified. The local ethics committee (Ethik Kommission der Ärztekammer Westfalen-Lippe und der Medizinischen Fakultät der Westfälischen Wilhelms-Universität, No. 2014-381-f-N) approved the study. Methods in this study were carried out in accordance with the current transplantation guidelines and the Declarations of Istanbul and Helsinki. Written informed consent was given by all participants at the time of transplantation for recording their clinical data.

We retrospectively analyzed all patients that underwent deceased donor RTx between January 2007 and December 2014 at the University Hospital Muenster. Living donor recipients and recipients aged < 18 years were excluded. Recipient and donor data were collected from the patients’ files. The following parameters were examined: patient and donor demographics, recipient body mass index (BMI), cause of end-stage renal disease (ESRD), number of prior kidney transplants, time on dialysis, degree of human leukocyte antigen (HLA)-mismatching, cold and warm ischaemia time. Weekend surgery was defined as the date of surgery being on a Saturday or Sunday and weekday surgery as the date of surgery being Monday to Friday.

Kidney donor profile index (KDPI) was calculated utilizing variables obtained from Eurotransplant network information system (ENIS)[15]. Recipients were divided into two categories: KDPI ≤ 85% and KDPI > 85%.

### Outcome measures

Primary outcome measures were patient, overall and death-censored graft survival. Patient survival was defined as time from RTx to death (from any cause) or last contact for patients alive. Overall graft survival was defined as time from RTx to death (from any cause), graft failure or last contact whatever occurred first. Graft failure was defined as reinitiation of dialysis treatment. Death-censored graft survival was defined as time from RTx to graft failure, analyzed in the framework of a multi-state model (see Statistical analysis). Secondary outcome parameters were incidence of delayed graft function (DGF, dialysis within the first week after RTx), serum creatinine and eGFR at 1 year after transplantation, frequency of biopsy-proven acute rejection episodes within the first year after RTx as well as the rate of complications requiring surgical intervention.

Whole blood was analyzed for creatinine (enzymatic assay; Creatinine-Pap, Roche Diagnostics, Mannheim, Germany) and renal function was determined by calculating the eGFR using the CKD-EPI equation.

Surgical complications requiring reoperation or interventional treatment were grouped into the following categories: Haemorrhagic complications (defined as any haematoma or bleeding that needed reintervention or blood transfusions), vascular complications (renal artery stenosis and vascular thrombosis), ureteral complications (urinoma, urinary leaks, ureteral obstruction with need for reintervention), wound complications (including impaired wound healing or dehiscence that needed surgical repair) and lymphoceles (with need for aspiration, drainage or surgical reintervention) [16,17]. Surgical complications were further classified by the day of occurrence post transplantation (≤ 30 days, 31–365 days and ≥ 366 days post transplantation). Details of surgical complications were related to the total number of complications in the respective group.

Length of initial hospital stay was determined from discharge reports after RTx.

As data regarding the acceptance of organ offers were not available until October 2015, the organ offer decline rate at our center was analyzed during a defined time period from October 2015 to January 2017.

### Statistical analysis

Statistical analysis was performed using IBM SPSS® Statistics 22 for Windows (IBM Corporation, Somers, NY, USA), SAS software, version 9.4 of the SAS System for Windows (SAS Institute, Cary, NC, USA) and R 3.2.5 [18].

Normally distributed continuous variables are shown as mean ± standard deviation (SD) and non-normally distributed continuous variables as median and interquartile range (IQR). Absolute and relative frequencies are given for categorical variables. Two independent groups of samples were compared using Student’s t-test for normally distributed outcome, Mann–Whitney U test for non-normal continuous outcome and Fisher's exact test for categorical outcome.

Logistic regression analysis was used to estimate the probability of surgical complication based on one or more predictor variables. Multivariable model building was performed using a stepwise variable selection procedure (inclusion: P-value of the score test ≤ 0.05, exclusion: P-value of the likelihood ratio test > 0.1). Variables included weekend transplant status, recipient age and sex, BMI, cause of ESRD, time on dialysis, prior kidney transplantation, cold ischaemia time, donor age and sex and KDPI. Results are presented as odds ratios (OR) with 95% confidence interval (95% CI) and p-value of likelihood ratio test. For non-selected variables in multivariable analyses, p-value of score test is given.

Survival analyses were based on a maximum follow-up of 3 years after RTx. Patient and overall allograft survival were estimated using Kaplan-Meier method [19], and groups were compared by log-rank test. Cox proportional hazards regression models [20] were built using a stepwise variable selection procedure to assess the association between weekend transplantation and survival while simultaneously adjusting for potential confounding factors (inclusion: P-value of the score test ≤ 0.05, exclusion: P-value of the likelihood ratio test > 0.1). Results are presented as hazard ratios (HR) with 95% confidence interval (95% CI) and p-value of likelihood ratio test. For non-selected variables in multivariable analyses, p-value of score test is given. To study both incidence of graft failure and the mortality rate, transition-specific Cox-regression was performed in the framework of an illness-death model with initial state “renal transplantation (RTx)”, transient state “graft failure” and absorbing state “death (from any cause)” using the mstate R package [21]. For the transition intensity to graft failure (death-censored survival), cause-specific hazard ratios with 95% confidence interval (95% CI) and p-value are given.

No adjustment for multiple testing was performed and analyses are regarded as explorative.

P-values ≤0.05 were considered as statistically noticeable.

## Results

### Patient cohort

Data was extracted from 580 deceased-donor kidney transplant recipients performed between January 2007 and December 2014 at our center. 416 (71.7%) patients underwent RTx on a weekday and 164 (28.3%) during a weekend. Baseline patient characteristics for donors and recipients and transplantation-associated parameters are shown in Table 1.

**Table 1:** Patient characteristics.

	Weekday RTx (n = 416)	Weekend RTx (n = 164)	p-value
**Age (years, mean ± SD)**	56.5 ± 12.4	54.7 ± 13.2	0.122^a^
**Male gender, n (%)**	252 (60.6)	98 (59.8)	0.851^c^
**BMI (kg/m**^**2**^**, mean ± SD)**	25.6 ± 4.2	25.7 ± 4.1	0.789^a^
**Diagnosis of ESRD, n (%)**			0.830^c^
Hypertension	39 (11.4)	14 (10.2)	
Diabetes	33 (9.6)	11 (8.0)	
Polycystic kidney disease	59 (17.3)	23 (16.8)	
Obstructive nephropathy	18 (5.3)	4 (2.9)	
Glomerulonephritis	94 (27.5)	38 (27.7)	
FSGS	15 (4.4)	8 (5.8)	
Interstitial nephritis	13 (3.8)	9 (6.6)	
Vasculitis	10 (2.9)	6 (4.4)	
Other	61 (17.8)	24 (17.5)	
**Time on dialysis (months, median (IQR))**	72.1 (41.1, 97.6)	73.2 (43.5, 99.8)	0.658^b^
**≥ 1 prior kidney transplant, n (%)**	42 (10.1)	22 (13.4)	0.303^c^
**Number HLA-A mismatch (0/1/2), n (%)**	153 (36.8) / 207 (49.8) / 56 (13.5)	67 (41.6) / 65 (40.4) / 29 (18)	0.101^c^
**Number HLA-B mismatch (0/1/2), n (%)**	116 (27.9) / 184 (44.2) / 116 (27.9)	41 (25.5) / 80 (49.7) / 40 (24.8)	0.512^c^
**Number HLA-DR mismatch (0/1/2), n (%)**	128 (30.8) / 172 (41.3) / 116 (27.9)	48 (29.8) / 75 (46.6) / 38 (23.6)	0.471^c^
**Cold ischaemia time (h, mean ± SD)**	10.9 ± 4.1	11.1 ± 4.3	0.648^a^
**Warm ischaemia time (min, mean ± SD)**	33.7 ± 7.4	32.8 ± 6.6	0.159^a^
**Donor age (years, mean ± SD)**	54.1 ± 16.7	53 ± 15.6	0.426^a^
**Male donor gender, n (%)**	197 (47.4)	81 (49.4)	0.712^c^
**KDPI > 85%, n (%)**	144 (34.8)	46 (28)	0.141^c^

Demographic characteristics of the study population by weekday-weekend transplant status. Results are presented as mean ± standard deviation (SD) or median and interquartile range (IQR), respectively, or as absolute and relative frequencies.

RTx = renal transplantation, BMI = body mass index, ESRD = end-stage renal disease, HLA = human leukocyte antigen, FSGS = focal segmental glomerulosclerosis, KDPI = Kidney donor profile index.

^a^ Student’s *t*-test

^b^ Mann-Whitney U test

^c^ Fisher’s exact test.

The two groups were similar with respect to all baseline characteristics that were analyzed.

### Patient survival, overall allograft survival and death-censored allograft survival

**Fig 1:**
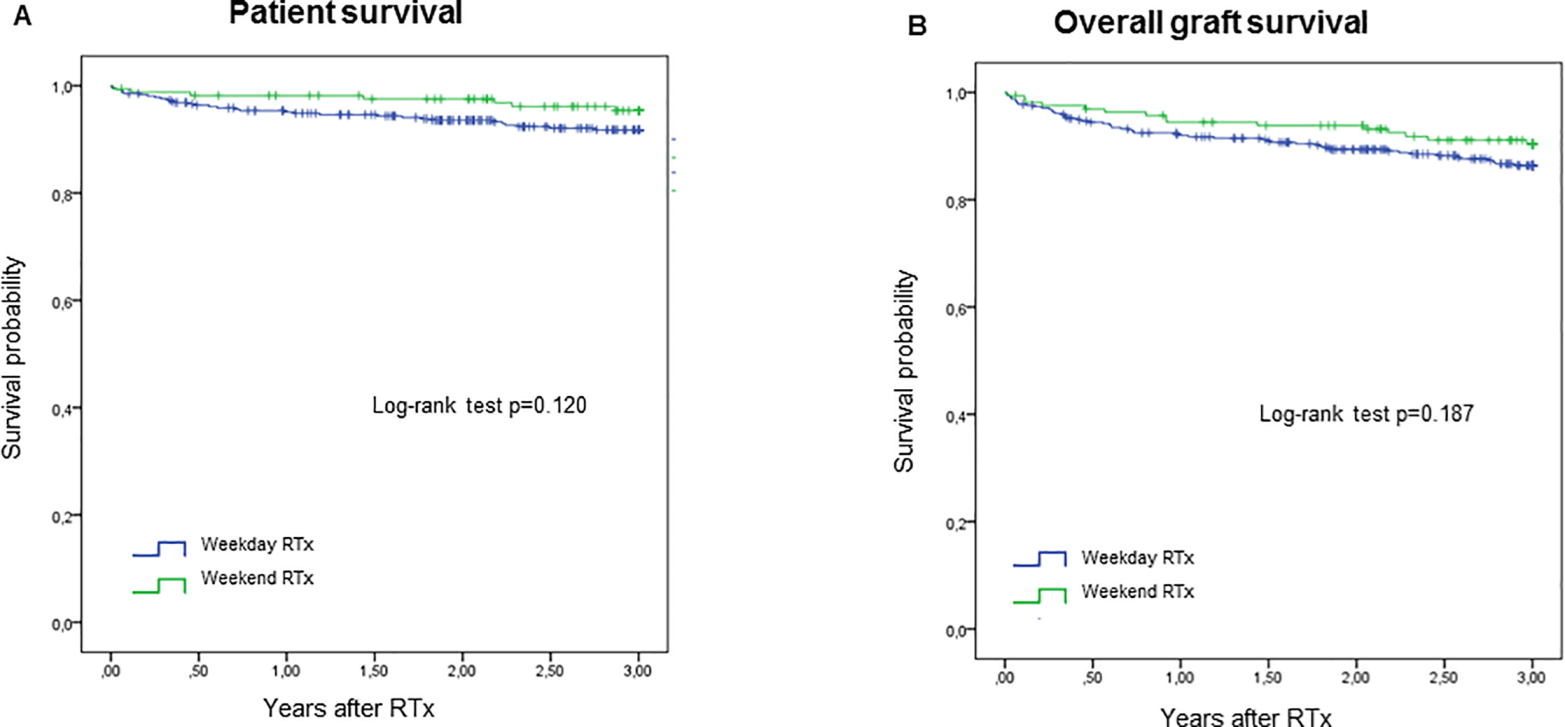
Kaplan-Meier curves for patient and overall graft survival. Patient survival (A) and overall graft survival (B) for weekday and weekend kidney transplantations. Survival rates of weekday (blue lines) and weekend (green lines) kidney transplant recipients were estimated by Kaplan–Meier method and compared by log-rank test.

**Fig 2:**
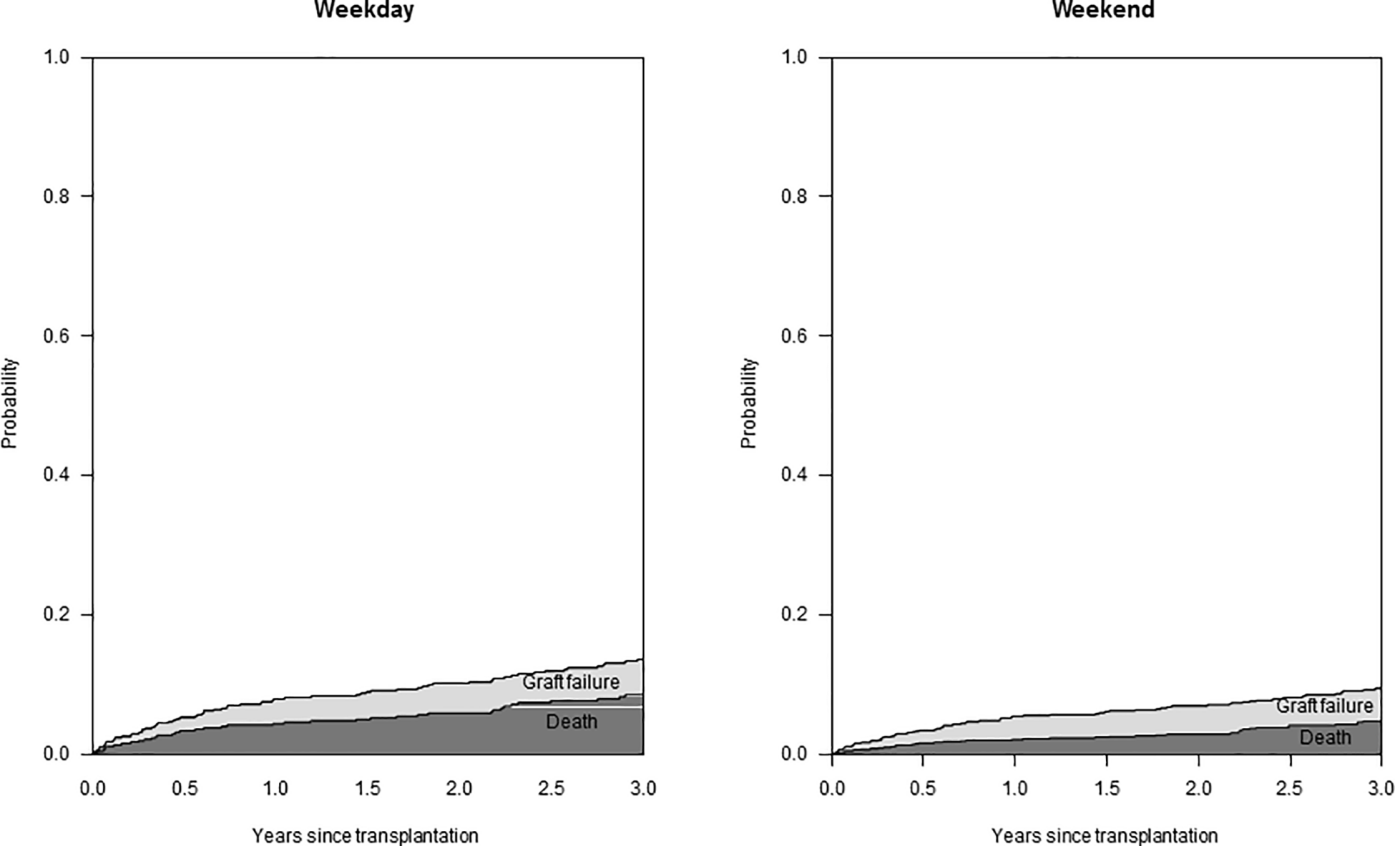
Transition probabilities by weekend transplant status derived from an illness-death model with initial state “renal transplantation (RTx)”, transient state “graft failure” and absorbing state “death (from any cause)”.

Kaplan-Meier curves for patient and overall allograft survival by weekend transplant status are shown in Fig 1. Transition probabilities from RTx to the states graft failure or death (from any cause) by weekend transplant status are shown in Fig 2. Survival parameters analyzed were comparable between patients transplanted on a weekend or on a weekday. 3-year patient survival was 92.3% and 95.7% (p = 0.120) and overall graft survival was 87.3% and 90.9% (p = 0.187) after weekday and weekend RTx, respectively. 3-year probability for graft failure following RTx was 4.7% and 5.1% (p = 0.874) after weekday and weekend RTx, respectively.

Univariable analyses did not reveal any statistically noticeable association between weekend transplant status and patient, death-censored and overall allograft survival. Similar results were obtained in (transition-specific) Cox regression analysis adjusted for potential confounders (Tables 2–4).

**Table 2:** Cox regression model for patient survival.

Parameters	Univariable		Multivariable	
	HR (95% CI)	p-value	HR (95% CI)	p-value
Weekend transplant status Weekend vs. Weekday (ref.)	0.528 (0.233–1.197)	0.103	-	0.217
Age (years)	1.045 (1.016–1.075)	0.001	1.057 (1.020–1.096)	0.001
Recipient gender Male vs. female (ref.)	1.369 (0.703–2.664)	0.348	-	0.226
Recipient BMI (kg/m^2^)	1.006 (0.934–1.084)	0.880	-	0.429
Cause of ESRD	-	0.129	-	0.483
Time on dialysis (months)	1.002 (1.001–1.002)	0.002	1.002 (1.001–1.003)	0.007
Prior kidney transplantation ≥ 1 vs. 0 (ref.)	1.790 (0.790–4.055)	0.191	-	0.067
Cold ischaemia time (hours)	0.966 (0.892–1.046)	0.384	-	0.719
Donor age (years)	1.016 (0.995–1.037)	0.130	-	0.525
Donor gender Male vs. female (ref.)	1.016 (0.542–1.904)	0.960	-	0.773
KDPI > 85% vs. ≤ 85% (ref.)	2.089 (1.115–3.916)	0.023	-	0.953

Univariable and multivariable analyses of patient and overall graft survival using Cox regression. Results are presented as hazard ratios (HR) with their 95% confidence interval (CI) and p-value of likelihood ratio test. For non-selected variables in multivariable analyses, p-value of score test is given.

HR = hazard ratio, CI = confidence interval.

**Table 3:** Cox regression model for overall graft survival.

Parameters	Univariable		Multivariable	
	HR (95% CI)	p-value	HR (95% CI)	p-value
Weekend transplant status Weekend vs. Weekday (ref.)	0.682 (0.384–1.209)	0.175	-	0.770
Age (years)	1.039 (1.017–1.061)	< 0.001	1.047 (1.021–1.075)	< 0.001
Recipient gender Male vs. female (ref.)	1.330 (0.805–2.198)	0.259	-	0.189
Recipient BMI (kg/m^2^)	0.997 (0.941–1.056)	0.914	-	0.131
Cause of ESRD	-	0.199	-	0.490
Time on dialysis (months)	1.001 (1.000–1.002)	0.036	1.001 (1.000–1.002)	0.054
Prior kidney transplantation ≥ 1 vs. 0 (ref.)	1.568 (0.822–2.990)	0.195	-	0.081
Cold ischaemia time (hours)	0.986 (0.930–1.044)	0.623	-	0.261
Donor age (years)	1.021 (1.005–1.037)	0.009	-	0.622
Donor gender Male vs. female (ref.)	0.737 (0.455–1.195)	0.213	-	0.120
KDPI > 85% vs. ≤ 85% (ref.)	2.318 (1.435–3.743)	0.001	-	0.209

Univariable and multivariable analyses of patient and overall graft survival using Cox regression. Results are presented as hazard ratios (HR) with their 95% confidence interval (CI) and p-value of likelihood ratio test. For non-selected variables in multivariable analyses, p-value of score test is given.

HR = hazard ratio, CI = confidence interval.

**Table 4:** Transition-specific Cox regression model for the transition to graft failure.

Parameters	Univariable		Multivariable	
	CHR (95% CI)	p-value	CHR (95% CI)	p-value
Weekend transplant status Weekend vs. Weekday (ref.)	0.940 (0.435–2.030)	0.873	-	0.432
Age (years)	1.037 (1.006–1.069)	0.015	-	0.699
Recipient gender Male vs. female (ref.)	1.301 (0.627–2.698)	0.474	-	0.473
Recipient BMI (kg/m^2^)	1.008 (0.928–1.095)	0.846	-	0.466
Cause of ESRD	-	0.269	-	0.219
Time on dialysis (months)	0.998 (0.977–0.998)	0.011	-	0.526
Prior kidney transplantation ≥ 1 vs. 0 (ref.)	1.512 (0.582–3.926)	0.418	-	0.331
Cold ischaemia time (hours)	0.991 (0.912–1.077)	0.829	-	0.182
Donor age (years)	1.032 (1.007–1.058)	0.008	-	0.534
Donor gender Male vs. female (ref.)	0.478 (0.227–1.010)	0.045	-	0.053
KDPI > 85% vs. ≤ 85% (ref.)	2.747 (1.353–5.576)	0.005	2.747 (1.353–5.576)	0.005

Univariable and multivariable transition-specific Cox regression in an illness-death model with initial state “renal transplantation (RTx)”, transient state “graft failure” and absorbing state “death (from any cause)”. Shown are results for the transition intensity to “graft failure” (death-censored survival). Results are presented as cause-specific hazard ratios (CHR) with their 95% confidence interval (CI) and p-value of likelihood ratio test. For non-selected variables in multivariable analyses, p-value of score test is given.

CHR = cause-specific hazard ratio, CI = confidence interval.

### Secondary outcomes

Secondary outcomes were not different between weekday and weekend RTx. Frequencies of DGF were similar in patients who underwent transplantation on a weekday (n = 97, 23.7%) or weekend (n = 38, 23.8%) (p = 1.000). Kidney function at 1 year after RTx as estimated by 1-year eGFR was equivalent (50.9 ± 19.9 and 49 ± 19.8 mL/min/1.73 m^2^, p = 0.335) and the frequency of patients experiencing ≥ 1 biopsy-proven acute rejection episode within the first year after transplantation was comparable after weekday (n = 69, 18.9%) and weekend (n = 31, 21.2%) RTx (p = 0.539) (Table 5).

**Table 5:** Frequencies of DGF, acute rejections within 1 year and 1-year eGFR.

	Weekday RTx	Weekend RTx	p-value
**DGF, n (%)**	97 (23.7)	38 (23.8)	1.000^b^
**≥1 biopsy-proven acute rejection within 1 year after RTx (%)**	69 (18.9)	31 (21.2)	0.539^b^
**1-year eGFR** **(CKD-EPI, ml/min/1.73m2, mean±SD)**	50.9 ± 19.9	49 ± 19.8	0.335^a^

Secondary outcomes compared by weekend transplant status. Groups were compared using Student’s t-test for normally distributed data and Fisher's exact test for categorical variables.

DGF = delayed graft function, eGFR (estimated glomerular filtration rate, CKD-EPI).

^a^ Student’s *t*-test

^b^ Fisher’s exact test.

### Surgical complications

**Fig 3:**
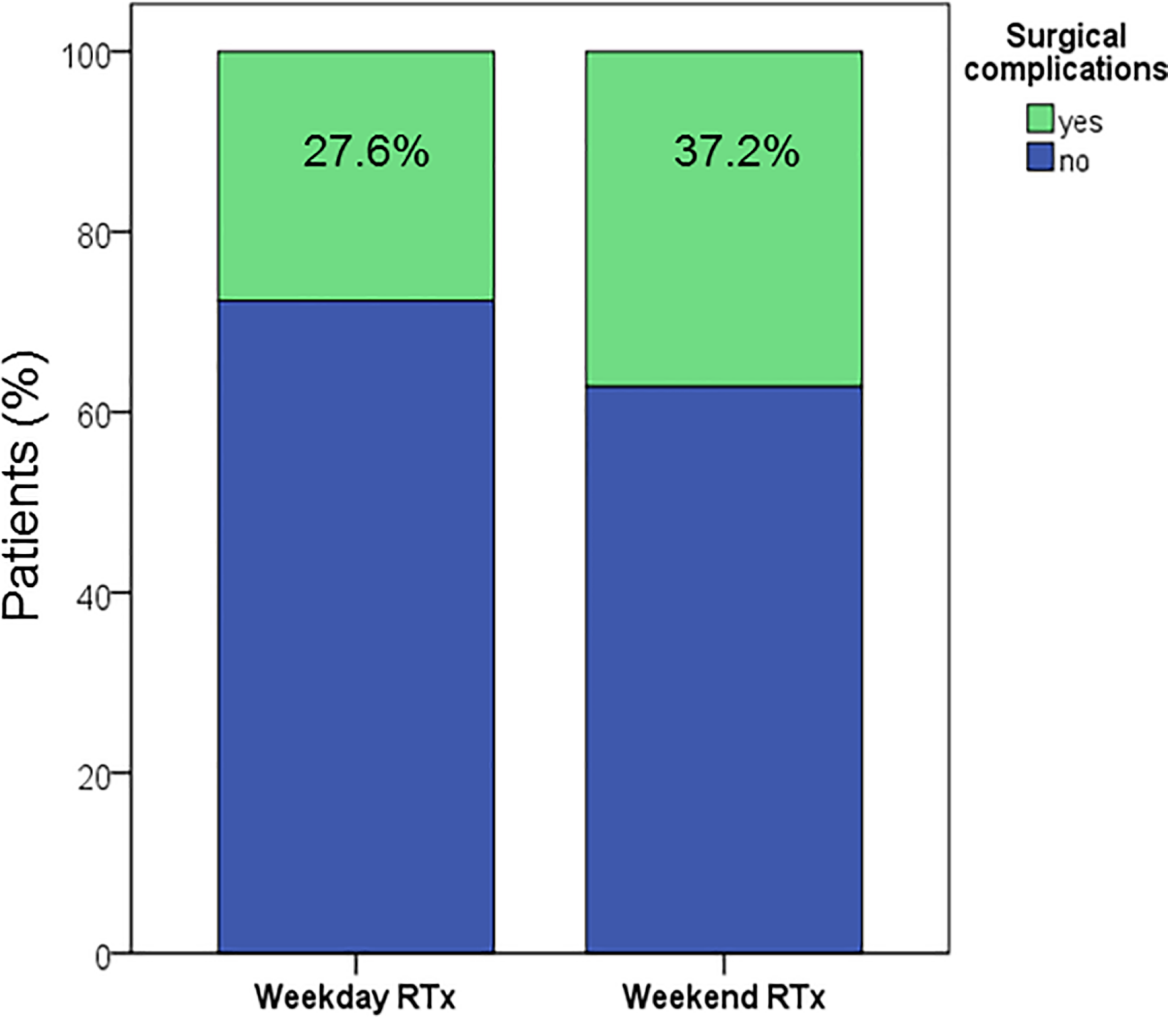
Frequency of surgical complications in weekday and weekend kidney transplant recipients.

Surgical complications requiring reoperation or interventional treatment occurred more often after weekend (n = 61, 37.2%) than after weekday transplantation (n = 115, 27.6%) and this difference was statistically noticeable in a multivariable analysis adjusted for potential confounders (OR 1.704 (95% CI 1.109–2.616), p = 0.016) (Fig 3, S1 Table). The most common surgical complications in both groups were haemorrhagic complications, followed by ureteral and wound complications. No differences were found regarding the date of occurrence post transplantation for any complication category.

### Duration of hospital stay

Median length of initial hospital stay after transplantation as determined by hospital discharge reports was similar in patients with transplants performed on weekdays and weekends (18.0 days (IQR 12.0–30.5) and 18.0 days (IQR 11.0–28.3), respectively, p = 0.731).

### Organ discard rate and donor quality

Due to missing previous data a period from October 2015 to January 2017 was defined as a representative observational period for analyzation of the organ offer decline rate. We did not detect any noticeable difference in the organ decline rate on weekends versus weekdays (57 (73.1%) vs. 156 (67.0%), p = 0.329).

KDPI was calculated to preclude a potential difference in donor quality between weekday and weekend donors. There was no difference in the frequency of patients with a KDPI > 85% between weekend and weekday organs (144 (34.8%) vs. 46 (28.0%), p = 0.141) suggesting that donor quality was equal in both groups.

All results remained consistent across all definitions of weekend status (Friday-Saturday or Sunday-Monday).

## Discussion

The ‘weekend effect’ describes increased adverse events and poorer outcomes for patients hospitalized on weekends. It has been demonstrated for various medical conditions such as myocardial infarction, ruptured aortic aneurysm, pulmonary embolism or stroke [6–8]. Limited resources in hospital staffing and delivery of care capacity as well as severity of illness of patients admitted to the hospital have been discussed to be responsible for this phenomenon. Due to the timing of organ availability RTx is regularly performed on weekends and, in contrast to other solid-organ transplants, not necessarily managed on intensive care units. Therefore, treatment of RTx patients is comparable to medical care in other inpatient wards and kidney transplant procedures may serve as a useful model to analyze the weekend effect.

Still, studies analyzing the weekend effect in the transplant setting are limited. In the context of liver transplantation Orman et al. have reported a small decrease in 1-year graft failure after weekend transplantation while patient survival was independent from the weekend transplant status [22]. Long-term patient and graft outcome was not assessed in this study. Regarding RTx two recent studies from the US and the UK have been published that did not find a weekend effect on patient and allograft survival in the short-term and long-term period after RTx, respectively [11,12].

However, other graft specific outcomes such as allograft function determined by eGFR, episodes of acute rejections or postoperative surgical complications have not been addressed so far. These are important limitations because complications can significantly influence the outcome after RTx and also constitute a relevant health care associated cost factor. Moreover, only the outcomes of organs that were procured and actually transplanted have been included in these studies without accounting for the impact of organ selection. The organ decline rate, however, might constitute a relevant confounder as Mohan et al. have recently shown that the organ acceptance rate in the US is not immune against the weekend effect, revealing a significant increase in the discard of organs procured on weekends with even lower Kidney Donor Profile Index scores [14,23].

In our study we confirmed the findings from the US and UK studies showing that the weekend effect does not affect patient and graft survival after RTx. Additional relevant secondary outcomes reflecting graft function and quality of medical care in the short-term and long-term post-transplant period such as DGF, 1-year eGFR, acute rejections, and length of hospital stay were not affected by weekend transplant status. A possible explanation for these findings might be that RTx, though often conducted during “off hours”, constitutes a standardized procedure. Its regular performance including the weekend days provides routine and experience in large transplant centers. As at our center 106 (mean, range 87–136) kidney transplants per year were performed during the study period, the complete process is comprised of a distinct course of procedures. Standardized pre- and postoperative care protocols are routinely followed by the involved staff irrespective of the day of the week.

Also, an increased alertness of the challenges and difficulties within the medical care system on weekends, in particular regarding the treatment of RTx recipients, might contribute to the comparable outcome between weekday and weekend RTx. The increased sensitivity towards this exceptional cohort of patients was stressed in a recent study by Manfredini et al. who showed that RTx recipients are not exposed to a higher risk of adverse outcome when admitted to the hospital on weekends compared to weekdays [24]. Moreover, patients on the waiting list are usually clinical stable, undergo regular investigations and are repeatedly seen by physicians and health care staff (at least patients on haemodialysis) in contrast to other patients that are admitted on weekends because of acute critical illness and emergencies. Differences in the severity of illness as well as the frequent medical screening which ensures a more accurate stratification of comorbidities might further contribute to the lack of a weekend effect after RTx [12].

In times of advanced immunosuppressive therapies the incidence of nonsurgery-related graft losses is decreased. The shortage of organ offers, however, has led to an increased use of marginal organs that often hold severe arteriosclerosis. Hence, a well-trained and experienced surgeon can significantly contribute to the allograft outcome [13]. In our cohort, postoperative surgical complications occurred more often after weekend RTx.

A possible explanation for the increased number of surgical complications after weekend RTx may be the surgical team’s schedule. On weekends the working hours for the surgical stuff exceed those during the week. Surgical outcome may decline when the primary surgeon has performed a high number of operations within a short period of time or is subjected to excessive duty hours and sleep deprivation. Fechner et al. have reported an increase in graft failure and surgical complications after RTx performed during nighttime compared to daytime [13]. However, these results were not confirmed by others [25,26]. The experience of the involved surgical explant and implant team might be a relevant influence factor [12,22]. However, this is unlikely to play a role at least at German or other Eurotransplant centers as strict guidelines exist that regulate organ procurement and ensure that only specialized transplant surgeons are allowed to perform these procedures. Also, in addition to the primary surgical team taking care of the weekend emergency cases, at our center there is always a dedicated transplant team on service which unexceptionally includes a senior surgeon independent from the day of the week. Indeed, this concept is subject of current debate in other countries such as England for example, where the introduction of the “seven day services”, guaranteeing a full-week consultant-led medical care in other medical disciplines, has been initiated by the Department of Health to overcome the weekend effect [12,27].

Other transplant team members than surgeons, such as anesthesiologists, might also contribute to peri- and postoperative performances. Though junior anesthesiologists might monitor the kidney recipient during the operation, we have a board certified anesthesiologist available in-house, which is alerted during critical phases such as reperfusion. Based on this permanent in-house availability of a board certified anesthesiologist during the weekend, we do not expect any change in the quality of care between weekdays and weekends. The surgical support staff, which can be an additional influence factor on postoperative outcome [22], is usually comprised of the same personnel throughout the week at our center thereby ensuring a consistent quality of care. All nurses involved in transplant procedures are not transplant-specific and either belong to regular weekend in-house teams or are part of additional on-call weekend teams. In both cases, there are no specific weekend nurses, and team members have to cover both weekend as well as weekday shifts periodically.

All patients are taken care of on an intermediate care unit after RTx surgery, where shift work is usual on both weekends as well as weekdays, meaning that also the post-operative care is comparable between weekend and weekday transplant recipients.

Personnel differences between weekends and weekdays like reduced or less-experienced medical staff are therefore unlikely to contribute to our finding. Due to limited data we were unable to investigate the organ decline rate during the observational period. However, data from a defined later period at our center was analyzed and did not reveal any difference regarding the decline rate on weekdays and weekends. This might again reflect the standardized processes, availability of medical resources and well-experienced medical stuff in the context of transplant-associated procedures at larger transplant centers. In this context, Mohan et al. observed that kidney utilization rates over the weekend were significantly higher at larger compared to smaller transplant centers on weekends [14]. The organ acceptance might be influenced by the accessibility of operating theatres at weekends. At our department, on weekends a fully equipped theatre is available 24 hours a day for emergency surgery. In case an organ is accepted, we also have an additional second theatre (including additional scrub nurses and anesthesiologists) in which we can perform emergency procedures as well as organ transplantations in parallel. Therefore, access to the operating theatre is not a limiting factor on weekends and should therefore have no influence on organ acceptance or outcomes following kidney transplantation.

To preclude a potential difference in donor quality we compared the (KDPI) between weekday and weekend donors. KDPI were similar in both groups suggesting that donor quality was not responsible for the observed outcomes. We recognize that a study of this nature has limitations because of its retrospective design and potential errors inherent to maintaining a single-center database. Moreover, due to the relatively small patient size inaccuracies in the data collection might affect the results though data acquisition was performed thoroughly to avoid inconsistency or entry errors. The analyses are based on the assumption that coding errors and missing data are stochastic.

Although we attempted to include as many relevant confounding parameters as possible there might still be residual factors that were not accounted for like experience level or previous working hours of the respective surgical team. Additionally, it would be interesting to evaluate the impact of daytime and nighttime transplantation which we did not perform in the present study.

The conventional definition of a weekend (Saturday–Sunday) may not be the right time span for analysis of weekend-associated adverse events. However, even when other definitions of weekend status were applied (Friday-Saturday or Sunday-Monday) the results remained consistent.

The findings of our study showing a lack of a weekend effect on patient and graft survival as well as on relevant secondary outcome parameters after RTx provide reassurance for the transplant community as well as for patients on the waiting list. Our study should mitigate concerns regarding safety and inferior outcome of kidney transplants performed on weekends. The standardized protocols and operationalized processes in centers routinely performing kidney transplants might contribute to this finding and may provide a model for other medical procedures that are performed on weekends to improve efficiency and outcomes.

It should of course be noted that outcome parameters assessed in this study are generally more affected by long-term factors such as recipients’ pre-existing conditions, graft quality, immunosuppressive regimen, and other long term post-transplant factors that exist far beyond just the specific day of surgery. As post-surgical complications are directly affected by the conduct and technical aspects of the operation performed and the immediate pre- and post-operative care, they might serve as a better endpoint in assessing for the existence of a “weekend effect” in the RTx population than long term graft outcome. Our finding that surgical complications were increased after weekend RTx requires further elaboration with special focus on other aspects of care that could be affected such as costs or patient satisfaction to fully assess the presence of a weekend effect in RTx. A comparison to other abdominal emergency surgeries is however difficult, as a unique feature of organ transplantation is the non-existing differentiation of elective vs. emergency procedures. This is an important consideration since surgical procedures performed at weekends are predominantly emergency ones and a potential bias in all studies evaluating a weekend effect in surgery is the higher ratio of emergency procedures at weekends.

Based on the results obtained here, preventive steps to avoid a weekend effect should include: a general awareness of the weekend effect, evaluation of any lack in professional personal, as well as an attempt to reduce the workload of transplant teams on weekends. Thus, when comparing weekend and weekday surgeries (e.g. cholecystectomy) a common explanation for an observed weekend effect is the severity of illness among weekend patients.

This is the first study that has looked at a possible weekend effect in a German transplant center. As transplantation practices as well as delivery of health care and resource availability may be different in other geographic regions or settings not fulfilling the criteria of a well-staffed transplant center, further studies are necessary to investigate possible weekday-weekend differences in these settings.

## Supporting information

S1 Table Univariable and multivariable binary logistic regression analysis for surgical complications. Results are presented as odds ratios (OR) with their 95% confidence interval (CI) and p-value of likelihood ratio test. For non-selected variables in multivariable analyses, p-value of score test is given.
OR = odds ratio, CI = confidence interval.
(DOCX)
