# Supplementary material for: Is there a "weekend effect" in kidney transplantation?
Source: PLoS One. 2017 Dec 28;12(12):e0190227. doi: 10.1371/journal.pone.0190227 (PMC5746275; doi:10.1371/journal.pone.0190227)
Supplement: S1 Table — Results are presented as odds ratios (OR) with their 95% confidence interval (CI) and p-value of likelihood ratio test. For non-selected variables in multivariable analyses, p-value of score test is given. OR = odds ratio, CI = confidence interval. (DOCX) [file pone.0190227.s001.docx]

**Supplementary Information**

**S1 Table.** Logistic regression model for predictors of surgical complications

| **Parameters** | **Univariable** | | **Multivariable** | |
| --- | --- | --- | --- | --- |
|  | OR (95% CI) | p-value | OR (95% CI) | p-value |
| Weekend transplant status Weekend vs. Weekday (ref.) | 1.550 (1.057 – 2.273) | 0.026 | 1.704 (1.109 – 2.616) | 0.016 |
| Age (years) | 1.017 (1.003 – 1.032) | 0.017 | 1.028 (1.010 – 1.045) | 0.001 |
| Recipient gender Male vs. female (ref.) | 0.928 (0.647 – 1.331) | 0.684 | - | 0.939 |
| Recipient BMI (kg/m^2^) | 1.014 (0.972 – 1.057) | 0.531 | - | 0.353 |
| Cause of ESRD | - | 0.327 | - | 0.551 |
| Time on dialysis (months) | 1.000 (0.999 – 1.001) | 0.565 | - | 0.572 |
| Prior kidney transplantation ≥ 1 vs. 0 (ref.) | 1.231 (0.711 – 2.132) | 0.462 | - | 0.995 |
| Cold ischaemia time (hours) | 1.045 (1.002 – 1.090) | 0.039 | 1.051 (1.003 – 1.102) | 0.038 |
| Donor age (years) | 1.012 (1.001 – 1.024) | 0.031 | - | 0.123 |
| Donor gender Male vs. female (ref.) | 1.564 (1.095 – 2.233) | 0.014 | 1.531 (1.026 – 2.285) | 0.036 |
| KDPI > 85 % vs. ≤ 85 % (ref.) | 1.362 (0.939 – 1.976) | 0.105 | - | 0.811 |
